# Patterns of Progression in Metastatic Estrogen Receptor Positive Breast Cancer: An Argument for Local Therapy

**DOI:** 10.1155/2017/1367159

**Published:** 2017-09-25

**Authors:** Patrick Kelly, Zhe Ma, Said Baidas, Rebecca Moroose, Nikita Shah, Roi Dagan, Eleftherios Mamounas, Justin Rineer

**Affiliations:** ^1^University of Florida Health Cancer Center-Orlando Health, Orlando, FL, USA; ^2^University of Central Florida College of Medicine, Orlando, FL, USA; ^3^Department of Radiation Oncology, University of Florida, Gainesville, FL, USA

## Abstract

**Purpose:**

Despite advances in endocrine therapy (ET), metastatic estrogen receptor positive breast cancer (BrCA) remains incurable. Though the mechanisms of resistance to ET have been studied extensively, the anatomic pattern of disease progression remains poorly characterized. The purpose of this study was to characterize the pattern of progression for patients receiving ET for metastatic BrCA.

**Methods:**

The records of 108 patients with metastatic BrCA who progressed on ET were reviewed. Progression was characterized as follows: diffuse progression, progression in greater than 3 sites; oligoprogression, progression in fewer than 3 sites with prior diffuse metastases; and oligometastatic disease with progression, progression in 3 or fewer sites with prior limited metastases.

**Results:**

Seventy-four patients (69%) displayed only diffuse disease progression. Conversely, 23 patients (21%) displayed oligoprogression and 11 patients (10%) displayed oligometastases with progression at least once in their disease course. Further analysis of the patients with oligoprogression suggested that in 14 patients the sites of progression would have been amenable to local therapy.

**Conclusion:**

Oligoprogressive disease occurs in a significant subset of patients with metastatic BrCA treated with ET. These patients with oligoprogressive disease may be eligible for local therapy, potentially obviating the need to change of systemic therapy.

## 1. Introduction

Though the treatment of metastatic breast cancer has evolved rapidly in the last 20 years, 40,000 people in the United States died of their disease in 2016 alone [1]. For the 60–70% of these patients with estrogen receptor positive metastatic breast cancer [2], the mainstay treatment is endocrine therapy (ET). This form of treatment targets the production of estrogen in the body or blocks the function of estrogen in the cancer cell directly [3]. Although ET is typically able to delay disease progression, almost invariably, patients will experience relapse of their disease. As disease progression is thought to represent the development of systemic resistance to ET, typically disease progression prompts a change in therapy often to a second-line ET [3]. Unfortunately, with time, disease progression will continue to occur, necessitating further changes in therapy often through multiple lines of endocrine therapy, targeted therapy, and cytotoxic therapy and eventually leading to the patient's death.

Although most patients with metastatic cancer will experience diffuse disease progression, with simultaneous growth at most of their sites of metastatic disease, in clinical practice, there appears to be a subgroup of patients in whom disease progression is more limited. The term oligoprogressive disease was coined to describe this form of limited disease progression. Different from the more widely appreciated concept of oligometastatic disease, in which patients appear to have a limited number of sites of metastatic disease [4, 5], patients with oligoprogressive disease often have many sites of metastatic disease, but only a limited number of sites develop resistance to therapy and progress [6, 7]. Though it has been described in several cancer types [8–13], oligoprogressive disease is best characterized in patients with epidermal growth factor receptor (EGFR) positive and anaplastic lymphoma kinase (ALK) positive non-small cell lung cancer (NSCLC) treated with receptor tyrosine kinase inhibitors (TKI) [7]. In this patient population, it appears that approximately 50% of patients with EGFR/ALK positive NSCLC will progress in as few as 1–4 sites. Moreover, multiple studies have suggested that the application of locally ablative therapies, such as stereotactic body radiation therapy (SBRT), to these sites of oligoprogression allows patients to remain on their TKI, resulting in prolonged progression-free survival (PFS) and improved overall survival (OS) [9, 10]. As a result, the use of ablative therapy for patients with oligoprogressive EGFR/ALK positive NSCLC has become an accepted standard of care [14].

Interestingly, though several parallels exist between EGFR/ALK positive NSCLC and endocrine receptor positive breast cancer, the phenomenon of oligoprogression in metastatic breast cancer has not been examined. Thus, the goal of this study was to systematically characterize the pattern of disease progression for patients with estrogen receptor positive metastatic breast cancer receiving ET. In doing so, we sought to determine whether a subset of these patients exhibit oligoprogressive disease and to analyze if these sites of oligoprogression might be amenable to ablative therapy.

## 2. Methods and Materials

After obtaining approval from the Institutional Review Board, the medical records of 512 patients with metastatic/recurrent breast cancer treated consecutively within the UF Health Cancer Center at Orlando Health between 2007 and 2013 were reviewed. From this patient population, 161 patients with ER and/or PR positive disease who received ET as part of the management of their metastatic breast cancer were identified. Of those, 108 patients who had at least three months of follow-up documented in the medical record and at least one episode of disease progression on ET were selected for analysis. The charts of the eligible patients were then reviewed for patient demographics, tumor characteristics, prior treatment history, endocrine therapy used, and overall survival. In addition, data regarding the pattern of progression at the time of ET failure including the number and location of the sites of disease progression were obtained through direct examination of the patient imaging studies performed at the time of progression and comparison with the patients' previous imaging studies. Diffuse progression was defined as progression in greater than 3 sites of disease and limited progression was defined as progression at 3 or fewer sites (existing or new). Oligoprogression was defined as progression in fewer than 3 sites with prior diffuse metastases (>6 sites of disease). Oligometastatic disease with progression was defined as progression in 3 or fewer sites with prior limited metastases (<6 sites of disease). For lesions in the brain, bone, lung, and liver, each radiologically identifiable lesion was considered one site of disease. For lesions in the lymph nodes, radiologic involvement of each echelon of the axillary, cervical, or mediastinal lymphatics was considered a single site of disease, even if there were multiple nodes noted in a given echelon. Lesions in or on the ipsilateral breast or chest wall were considered a single site of disease, even when multiple lesions were visible radiographically or clinically. Leptomeningeal disease, malignant pleural effusions, and cutaneous involvement outside the ipsilateral breast or chest wall were considered diffuse disease.

### 2.1. SBRT Eligibility

In order to determine if the patients with limited progression were candidates for ablative therapy, two independent physicians with experience in SBRT reviewed each of the cases of limited progression. The physicians sought to determine if the sites of progression were eligible for SBRT. Eligible sites included lesions in the lung, liver, and bone where the investigating physicians determined whether it would be possible to safely treat all of the sites of progression using the dose and fractions schema of the phase II/III trial NRG-BR002: a Phase IIR/III Trial of Standard of Care Therapy with or without Stereotactic Body Radiotherapy (SBRT) and/or Surgical Ablation for Newly Oligometastatic Breast Cancer (NCT02364557). Dose and fractionation schema to the relevant anatomic sites range from 30–45 Gy in 3 fractions to 50 Gy in 5 fractions. Stereotactic radiation therapies to progressing sites of disease in the brain were evaluated per standard guidelines.

### 2.2. Follow-Up and Statistical Analysis

The median follow-up time for patients in this study from time of metastatic disease diagnosis was 31 months (range, 6–103 months). During the period of study, per institutional practice, most patients underwent regular reimaging on an every-2–4-month basis or when prompted by symptoms. Time to progression was calculated from the date of initiation of ET to the date of imaging study demonstrating disease progression. Overall survival (OS) curves were calculated using the Kaplan-Meier method, and tests of significance were based on the log-rank statistic. Differences between proportions for categorical variables were analyzed using a two-sided Fisher's exact test. All data were computed using SPSS 15.0 for Windows (SPSS, Chicago IL). A *p* value of less than 0.05 was accepted as significant. Figures for publication were generated using Graph Pad Prism 5.0 (Graph Pad Software, La Jolla, CA).

## 3. Results

### 3.1. Patient and Treatment Characteristics

**Figure 1:**
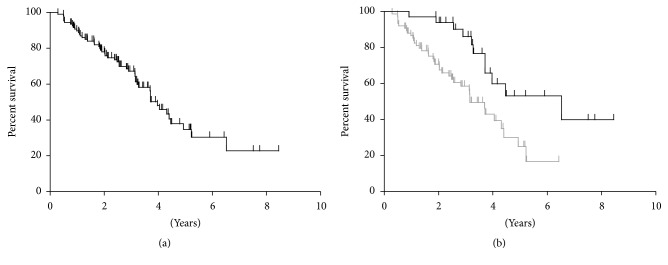
Kaplan-Meier plots of the overall survival from time of diagnosis of metastatic disease for (a) the entire 108-patient study population and (b) the study population divided into a cohort of patients who only experienced diffuse disease progression (gray line) and a cohort patients who experienced oligoprogression or oligometastases with progression at least once in their disease course.

In total, 108 patients were identified who received endocrine therapy for estrogen receptor positive metastatic breast cancer at the UFCC at Orlando Health between 2007 and 2013. The majority of these patients (64%) had previously been treated for localized breast cancer prior to being diagnosed with metastatic disease. Of these patients, 65% had received cytotoxic therapy and 64% had received at least 3 months of hormone therapy prior to being diagnosed with metastatic disease. The patients' tumors were predominantly ER+/PR+/Her2− (68%), with smaller percentages of patients harboring ER+/PR−/Her2− (13%), ER+/PR+/Her2+ (11%), and ER+/PR−/Her2+ (8%) disease (Table 1). Most patients underwent PET/CT scan at the time of diagnosis of metastatic disease (82%), with the remainder receiving at minimum CT of the chest, abdomen, and pelvis and a bone scan. Greater than 6 sites of disease were noted at the time of diagnosis of metastatic disease in 72% of patients, with the most common sites of disease being bone, locoregional, lung/mediastinum, and liver in 64%, 39%, 34%, and 31% of patients, respectively. The median overall survival for the entire cohort from time of diagnosis with metastatic disease was 3.9 years, with 34% of patients alive at 5 years (Figure 1(a)).

Altogether, the cohort of 108 patients studied received 195 courses of endocrine therapy in which disease progression was noted. Fifty-seven patients (53%) received 1 course of therapy, 18 patients received 2 courses (17%), and 33 patients (30%) received more than 2 courses of therapy (Table 2). The most common form of endocrine therapy utilized by course was aromatase inhibitor (AI) alone (58%), followed by fulvestrant (30%), AI with a gonadotropin releasing hormone agonist (23%), and tamoxifen (17%). Other therapies included AI with everolimus (11%) and AI with fulvestrant (9%). In addition, endocrine therapy was supplemented with Her2 targeted therapy in 19% of the courses and with a bisphosphonate or denosumab in 77% of the courses. At the time of progression, salvage therapy was offered to 89% of cases, with 61% receiving further endocrine therapy and 39% receiving cytotoxic therapy. In addition, palliative radiation therapy was delivered in 8% of cases of when progression was noted.

### 3.2. Patterns of Failure and Outcomes

For the 108 patients included in this study, we found 195 instances of disease progression on ET that prompted a change in therapy. Progression was characterized as follows: diffuse progression, progression in greater than 3 sites; oligoprogression, progression in fewer than 3 sites with prior diffuse metastases (>6 sites of disease); and oligometastatic disease with progression, progression in 3 or fewer sites with prior limited metastases (<6 sites of disease). Examination of the patterns of failure revealed diffuse progression in 150 courses (77%), oligoprogression in 28 courses (14%), and oligometastases with progression in 18 courses (9%). On a per patient basis, most patients (69%) displayed only diffuse disease progression, with a smaller percentage of patients displaying oligoprogression (21%) or oligometastases with progression (10%) at least once in their disease course. Though these patients with oligoprogression or oligometastases with progression were similar in age and prior treatment to those patients who progressed diffusely, the patients who progressed diffusely were more likely to be Her2− (84% versus 74%, *p* < 0.04), have more than 6 sites of disease at metastatic diagnosis (80% versus 56%, *p* < 0.04), and have boney metastases (72% versus 47%, *p* < 0.02) (Table 3). Additionally, though the median time to progression with each course of ET appeared similar between the two groups (Table 2), the overall survival of the patients that displayed diffuse progression was significantly shorter than the patients with oligoprogression or oligometastases with progression (median survival 3.1 years versus 6.5 years, *p* < 0.03) (Figure 1(b)).

**Figure 2:**
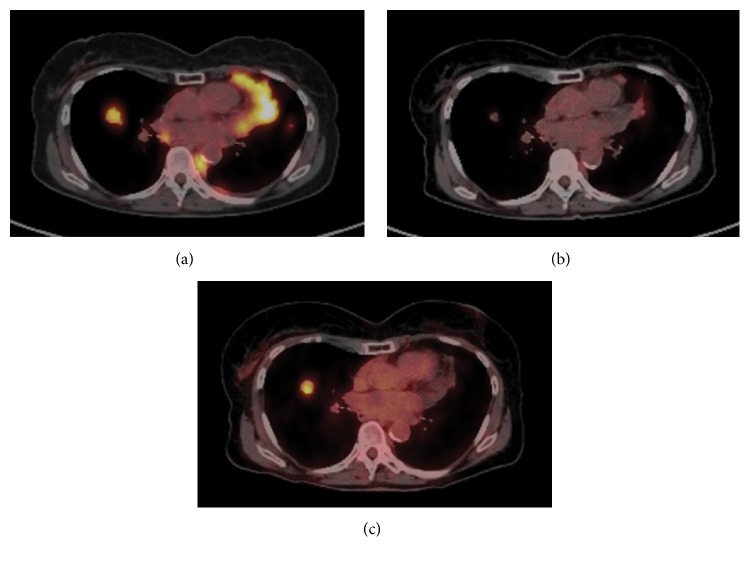
Representative example of a patient with oligoprogressive metastatic breast cancer. (a) PET scan of the central chest at the time of diagnosis of metastatic disease. (b) PET of the same area after 8 months of fulvestrant demonstrating resolution of the FDG avid disease. (c) Surveillance PET scan 6 months later demonstrating solitary site of progressive disease in the right lung. No other evidence of progressive disease was noted in this patient.

Of the 23 patients with oligoprogressive disease, 11 patients had only one site of progressive disease, 10 patients had two sites of progressive disease, and 2 patients had three sites of progressive disease. The most common sites of oligoprogression were bone (*n* = 9), liver (*n* = 5), locoregional (*n* = 3), and lung (*n* = 2) (Figure 2). In addition, mixed locoregional and boney metastases were noted in two patients and one patient developed brain metastasis. Unfortunately, given the limited number patients in this study, no treatment or demographic factors were identified that were specifically associated with oligoprogression.

### 3.3. SBRT Eligibility

As noted above, ablation of sites of oligoprogression has demonstrated clinical benefit in other malignancies. Therefore, while characterization of the pattern of progression for patients with metastatic estrogen receptor positive breast cancer was the primary aim of this study, we also sought to identify if any of the patients with apparent oligoprogressive disease would have been amenable to ablative therapy. Stereotactic body radiation therapy (SBRT) was selected as the modality to be assessed as it has demonstrated efficacy in other malignancies, is noninvasive, and has documented low rates of significant morbidity. Of the patients with oligoprogressive disease, 14 (61%) had disease deemed amenable to ablative therapy (Supplemental Table 1 in Supplementary Material available online at https://doi.org/10.1155/2017/1367159). The patients with disease amenable to ablation included 7 patients with spinal metastasis, 4 patients with liver metastasis, 2 patients with lung metastasis, and 1 patient with brain metastasis. Seven patients had one lesion, 5 patients had 2 lesions, and 2 patients had 3 lesions.

## 4. Discussion

Metastatic estrogen receptor positive breast cancer is a heterogeneous disease in which overall survival can vary from a few months to several years. In order to better understand this heterogeneity, we performed a detailed analysis of the pattern of failure of patients with metastatic estrogen receptor positive breast cancer receiving ET. This analysis suggested that while most patients progress diffusely, in a subset of patients, limited disease progression, oligoprogression, may occur and that, for some patients, local ablative therapy may be an alternative treatment option.

In the current study, of the 108 patients examined, oligoprogressive disease was noted in only 23 patients (21%). Further, only a subset of 14 of these patients, or 13% of the overall population, appeared amenable to ablative therapy. As such, in both incidence and amenability to ablative therapy, the percentage of patients with oligoprogressive metastatic estrogen receptor positive breast cancer appears significantly lower than that seen in ALK/EGFR positive NSCLC. Nevertheless, given the small number of patients with ALK/EGFR positive NSCLC, it is likely that the worldwide incidence of oligoprogressive disease is significantly greater in the setting of metastatic breast cancer. In addition, in order to determine if the patients with oligoprogressive disease in the current study were amenable to local therapy, we conservatively chose to include patients who had <3 sites of disease and that were all amenable to treatment by the metrics delineated in the ongoing NRG BR002 trial. As such, the percentage of patients with metastatic breast cancer who progress on hormone therapy that would benefit from local therapy may be higher than determined in this study. For example, patients with local recurrences/regional disease progression were not considered candidates for ablative therapy though it is likely that some of these patients would be amenable to definitive radiation or surgery. As such, though the absolute incidence is low, the impact of oligoprogressive disease metastatic estrogen receptor positive breast cancer may be significant.

There are a number of limitations to this study related to its retrospective design. The eligibility criteria used for patient selection may have biased the cohort towards patients with better prognosis. This is evidenced by comparison of the overall survival seen in the current study to that seen on recently completed studies of first-line therapy for hormone receptor positive breast cancer [15, 16]. As such, the incidence of oligoprogressive disease in this study may be inflated compared to the population of patients with estrogen receptor positive metastatic breast cancer in general. Second, as follow-up on the study was not uniform, it is possible that oligoprogressive events were missed or that some of the oligoprogressive events represented early diffuse progression. However, as 86% of the patients had at minimum a PET/CT scan at the time of progression, characterization of the individual progressive events was fairly robust.

In conclusion, this study demonstrates that a subset of patients with metastatic estrogen receptor positive breast cancer receiving ET undergo changes in therapy as a result of progression in a limited number of disease sites. Further, analysis of these sites of oligoprogression indicates that, in approximately half of these patients, the sites of progression would have been amenable to local ablative therapy. Taken together, these findings suggest that the application of local therapy to the sites of oligoprogressive disease in estrogen receptor positive metastatic breast cancer may obviate the need for therapeutic change, potentially prolonging the duration of endocrine therapy effectiveness and, ultimately, survival. Nevertheless, given the respective nature of this study, this conclusion cannot be confirmed without further research. A prospective trial is currently under development at our institution to test this hypothesis.

## Supplementary Material

Supplemental Table 1: Patients with oligoprogressive, treatment characteristics and description of disease progression.

## Figures and Tables

**Table 1:** Treatment characteristics by course of endocrine therapy.

	Entire cohort	Diffuse progression	Limited progression (<3 sites)		
			Entire cohort	Oligoprogression	Oligometastatic + progression
Courses, *n*	195	150	45	28	18

Type of therapy by course, *n* (%)					
Aromatase inhibitor	77 (39%)	58 (39%)	19 (42%)	13 (48%)	6 (33%)
AI + GNRH agonist	29 (15%)	23 (15%)	6 (13%)	2 (7%)	4 (22%)
AI + everolimus	13 (7%)	11 (7%)	2 (4%)	2 (7%)	0 (0%)
AI + fulvestrant	14 (7%)	9 (6%)	5 (11%)	4 (15%)	1 (6%)
Fulvestrant	38 (19%)	30 (20%	8 (18%)	3 (11%)	5 (28%)
Tamoxifen	22 (11%)	17 (11%)	5 (11%)	3 (11%)	2 (1%)
Other	2 (1%)	2 (1%)	0 (0%)	0 (0%)	0 (0%)

Additional treatments, *n* (%)					
Bisphosphonate use	102 (52%)	77 (51%)	25 (56%)	19 (70%)	6 (33%)
Her2 target therapy	37 (19%)	19 (13%)	16 (36%)	7 (26%)	9 (50%)

Mean time to progression (per course)	8 (1–61) months	8 (1–38) months	9 (2–61) months	12 (2–30) months	6 (2–61) months

AI: aromatase inhibitor; GNRH: gonadotropin-releasing hormone.

**Table 2:** Patient, tumor, and treatment characteristics.

	Entire cohort	Diffuse progression	Limited progression (<3 sites)		
			Entire cohort	Oligoprogression	Oligometastatic + progression
Patients, *n*	108	74	34	23	11

Age at diagnoses, median (range)	55 (22–82) years	54 (22–80) years	57 (26–82) years	59 (39–82) years	52 (26–76) years
Age at mets	55 (22–84) years	55 (22–84) years	59 (28–82) years	60 (41–82) years	55 (28–76) years

Phenotype, *n* (%)					
ER(+)/PR(+)/Her2(−)	73 (68%)	54 (73%)	19 (56%)	17 (74%)	2 (18%)
ER(+)/PR(−)/Her2(−)	14 (13%)	8 (11%)	6 (18%)	3 (13%)	3 (27%)
ER(+)/PR(+)/Her2(+)	12 (11%)	7 (9%)	5 (15%)	1 (4%)	4 (36%)
ER(+)/PR(−)/Her2(+)	9 (8%)	5 (7%)	4 (12%)	2 (9%)	2 (18%)

Time to metastases					
Median (range)	1 (0–99) months	1 (0–99) months	10 (0–49) months	4 (0–49) months	10 (0–30) months
≤6 months from initial Dx, *n* (%)	63 (58%)	46 (62%)	17 (50%)	13 (57%)	4 (36%)
>6 months from initial Dx, *n* (%)	45 (42%)	28 (38%)	17 (50%)	10 (43%)	7 (64%)

Extent of metastases, *n* (%)					
≤6 sites of disease	30 (28%)	15 (20%)	15 (44%)	4 (17%)	11 (100%)
>6 sites of disease	78 (72%)	52 (70%)	19 (56%)	19 (83%)	0 (0%)

Sites of disease, *n* (%)					
Bone	69 (64%)	53 (72%)	16 (47%)	12 (52%)	4 (36%)
Local/regional	42 (39%)	34 (46%)	7 (21%)	4 (17%)	3 (27%)
Lung/mediastinum	37 (34%	27 (36%)	10 (29%)	9 (39%)	1 (9%)
Liver	34 (31%)	22 (30%)	12 (35%)	8 (35%)	4 (36%)
Brain	4 (4%)	3 (4%)	1 (3%)	1 (4%)	0 (0%)
Other	9 (8%)	7 (9%)	2 (6%)	2 (9%)	0 (0%)

Prior chemotherapy, *n* (%)	45 (42%)	24 (32%)	17 (50%)	11 (48%)	6 (55%)
Prior endocrine therapy, *n* (%)	44 (41%)	28 (38%)	16 (47%)	10 (43%)	6 (55%)

Rounds of endocrine therapy, *n* (%)					
1	57 (53%)	47 (64%)	10 (29%)	5 (22%)	5 (45%)
2	18 (17%)	8 (11%)	10 (29%)	8 (35%)	2 (18%)
3	29 (27%)	17 (23%)	12 (35%)	9 (39%)	3 (27%)
4	4 (4%)	2 (3%)	2 (6%)	1 (4%)	1 (9%)

ER: estrogen receptor; PR: progesterone receptor; Her2: Her2neu.

**Table 3:** Univariate analysis of clinical factors potentially associated diffuse disease progression.

	Patients with limited disease progression^*∗*^	Patients with diffuse disease progression	*p*
Age			
<65	56%	60%	0.95
≥65	44%	40%	
Phenotype			
Her2(+)	26%	16%	<0.04
Her2(−)	74%	84%	
Time to metastasis			
≤6 months from initial Dx	50%	58%	0.85
>6 months from initial Dx	50%	42%	
Longest time to progression			
≤12 months	50%	42%	0.85
>12 months	50%	58%	
# of metastatic sites			
≤6 sites of disease	44%	20%	<0.04
>6 sites of disease	56%	80%	
Disease site			
Bone	47%	70%	<0.02
Other	53%	30%	

^*∗*^Patients displaying oligoprogression or oligometastases with progression at least once in their disease course.

## Abbreviations

ET: Endocrine therapy
ER: Estrogen receptor
PR: Progesterone receptor
SBRT: Stereotactic Body Radiotherapy
EGFR: Epidermal growth factor receptor
ALK: Anaplastic lymphoma kinase
NSCLC: Non-small cell lung cancer.
